# ‘Cut from the same cloth’: Shared microsatellite variants among cancers link to ectodermal tissues-neural tube and crest cells

**DOI:** 10.18632/oncotarget.4194

**Published:** 2015-06-17

**Authors:** Enusha Karunasena, Lauren J. Mciver, Jasmin H. Bavarva, Xiaowei Wu, Hongxiao Zhu, Harold R. Garner

**Affiliations:** ^1^ Virginia Bioinformatics Institute, Medical Informatics and Systems Division, Virginia Tech, Blacksburg, VA 24061, USA; ^2^ Department of Statistics, Virginia Tech, Blacksburg, VA 24061, USA

**Keywords:** microsatellite, glioma, medulloblastoma, melanoma, neuroblastoma

## Abstract

The pluripotent cells of the embryonic ectodermal tissues are known to be a precursor for multiple tumor types. The adaptability of these cells is a trait exploited by cancer. We previously described cancer-associated microsatellite loci (CAML) shared between glioblastoma (GBM) and lower-grade gliomas. Therefore, we hypothesized that these variants, identified from germline DNA, are shared by cancers from tissues originating from ectodermal tissues: neural tube cells (NTC) and crest cells (NCC). Using exome sequencing data from four cancers with origins to NTC and NCC, a ‘signature’ of loci significant to each cancer (*p*-value ≤ 0.01) was created and compared with previously identified CAML from breast cancer. The results of this analysis show that variant loci among the cancers with tissue origins from NTC/NCC were closely linked. Signaling pathways linked to genes with non-coding CAML genotypes revealed enriched connections to hereditary, neurological, and developmental disease or disorders. Thus, variants in genes from tissues initiating from NTC/NCC, if recurrently detected, may indicate a common etiology. Additionally, CAML genotypes from non-tumor DNA may predict cancer phenotypes and are common to shared embryonic tissues of origin.

## INTRODUCTION

Neural Tube & Crest Cells: During embryogenesis the neural tube gives rise to the glial cells of the central nervous system (CNS) and the pluripotent cells of the neural crest migrate extensively throughout the embryo and give rise to a number of differentiated cell types, including peripheral and enteric neurons, glia, melanocytes, Schwann cells, and cells of the craniofacial skeleton and adrenal medulla [1, 2]. After primary development ceases, neurons become post-mitotic and only a small compartment of stem cells remain, whereas glial cells retain the ability to proliferate throughout life. In this context, it is perhaps not surprising that most adult neurological tumors are of glial origin [3]. The classification of brain tumors is based on the predominant cell type(s), which is generally determined by morphological and immunohistochemical criteria. Therefore, improved insight into the interrelated hereditary, genetic, and genomic traits shared by these cancers might be relevant for the identification of tissue/tumor specific cancer prognosis and therapies.

DNA Microsatellite Repeat Loci: Variable tandem repeat loci, including microsatellites (MST), are causative or linked with many hereditary neural diseases and cancers most notably hereditary nonpolyposis colorectal cancers [4]. Modifications to coding and non-coding regions of these short repetitive sequences can result in mutations leading to modified mRNA and protein synthesis. Thus, alterations to these unique repetitive regions are associated with alternative mRNA splicing, microRNA synthesis, modified binding sites for transcription factors and changes in gene expression [4–6]. Microsatellite loci are identified as 6-12 nucleotides in length, consisting of monomeric, di, tri, and up to heptameric motifs [5, 7]. Non-coding repeat sequences promote changes in replication and transcription binding machinery which can also contribute to changes in molecular and biological functions that propagate disease [4]. Repeat containing loci are highly vulnerable to genomic variation and they represent as much as 3% of the human genome; twice the size of the coding region [8, 9]. These simple sequences have attracted attention because expansion of tri-nucleotide repeat sequences are important contributors to over 40 different neurological disorders including Fragile X, Huntington's and Parkinson's disease [4]. Thus, changes to microsatellite sequences yield phenotypes and contribute to diseases.

We have developed a microsatellite genotyping algorithm that is highly accurate (96.5%) and has been successfully used to identify breast and brain cancer-linked DNA, MST variants in addition to variants identified in response to cell stressors and aging [10–13]. From predetermined histopathology classification of tumor and germline sequencing data we identified non-coding microsatellite loci that differentiate GBM from lower-grade gliomas (LGG) [14]. These data and others suggest it may be possible to identify a unique ‘signature’, for cancers of NTC/NCC lineage. We therefore hypothesized that the identification of these cancer-specific variant loci from germline DNA would indicate a common embryonic tissue of origin and that these variant loci could serve as targets for the development of powerful combination therapeutics and foster a novel understanding in cancer etiology.

## RESULTS

We analyzed tumors whose tissues originate from embryonic ectodermal tissues (NTC and NCC) with breast cancer, a non-NTC/NCC tissue, to identify the extent of shared CAML genotypes. First, we identified no significant differences in shared CAML genotypes of MEL, GBM, LGG, and MB (cancers with tissues originating from NTC/NCC) from each other. Whereas: GBM, LGG, and MEL compared with BC all demonstrated statistically significant differences (*p* < 0.05). MB when compared with BC and LGG showed statistically significant overlap (*p* < 0.05), see Table 1. MB had the smallest sample population and signature CAML set, with a larger sample cohort a more robust signature could further extricate loci specific to this disease. In the 3-set overlap analysis, shared CAML genotypes between the cancers originating from NTC /NCC lineage with BC were 0 or 1 locus (see Table 1). Genotypes from loci identified from tumors with NTC/NCC lineage showed multiple shared CAML genotypes (see Table 1); the most common locus identified in MEL, GBM, LGG, and MB was in an intron of *PSME* (15:63040517-63040532). The second most frequently shared locus was in an intron of *LAMP1* (13:115002098-115002110). Unique to all of the brain cancers (GBM, LGG, and MB) was a locus in the intron of *FUBP3* (9:133498230-133498244). The most common CAML identified in BC that were also in neural tissue cancers included the following genes: *TLN2* (in MEL and MB); *KIF1B* and *NCOR1* (MEL and LGG). Interestingly, all seven CAML common to BC and GBM were unique to this relationship and were not identified in the other comparisons tested against BC.

**Table 1:** CAML Genotypes Shared by NTC/NCC Lineage Cancers & BC

Variant Microsatellite Loci in Individual Cancers			
Cancer	Sample Population (*n*)	Significant Genotypes	Signature CAML Genotypes (FDR Corrected)
GBM	252	178	48
LGG	136	145	42
MEL	149	157	68
MB	51	58	12
BC	656	242	52

Signature CAML Genotypes Shared by Multiple Cancers		
3-Way Comparison of Cancers	Shared CAML	Significance (*p*-value)
MEL v. GBM v. LGG	4	0.792
MEL v. GBM v. MB	1	1.000
MEL v. BC v. GBM	0	0.011*
LGG v. BC v. GBM	0	0.023*
LGG v. MB v. MEL	2	0.986
LGG v. GBM v. MB	2	1.000
MB v. BC v. LGG	0	0.037*
MB v. BC v. MEL	1	0.175

Signature CAML Genotypes Shared between Cancers	
Pair-Wise Comparison of Cancers	Number of Shared CAML
MEL v LGG	23
MEL v GBM	7
MEL v MB	6
MEL v BC	3
LGG v GBM	6
LGG v MB	4
LGG v BC	2
GBM v MB	3
GBM v BC	7
MB v BC	1

Described for each disease cohort- Glioblastoma (GBM), lower grade glioma (LGG), melanoma (MEL), medulloblastoma (MB), and breast cancer (BC) are the number of samples (n) analyzed to identify significant microsatellite loci, and those loci with genotypes which form a signature of cancer-associated allelic pairs based on false discovery rate correction. Further described are the analyses of FDR corrected, signature loci shared between different cancers. Cancers compared in sets of 3 are described with a *p*-value (*p* < 0.05) and significance (*). Lastly, the number of shared signature CAML between any two cancers is described.

In a separate analysis, signature loci common to any pair-wise comparison was used to identify the number of mutual CAML from either of two cancers. Results demonstrate: MEL, LGG, and MB shared the most CAML loci; CAML from LGG and MEL compose most of the loci found in the MB signature (see Tables 1 and 2). The least common loci were between (adult brain cancer) GBM and (childhood brain cancer) MB, see Table 2. Similar percentages of mutual CAML were identified when each of the cancers were analyzed with GBM, including LGG. LGG, MB, and MEL exhibited more variability in two-way comparisons as opposed to pair-wise comparisons with BC or GBM.

**Table 2:** Shared Signature Microsatellite Loci

Percentage of Shared Signature Variant Microsatellite Loci Between Cancer Comparisons				
	GBM	LGG	MEL	MB
**BC**	9.3%	8.5%	9.4%	11%
**GBM**	-	12%	14%	0.2%
**LGG**	-	-	26%	33%
**MEL**	-	-	-	32%

Compared are four cancers with linkage to NTC/NCC lineage (GBM, LGG, MEL, and MB) compared with breast cancer (BC). Described is the relative percentage of signature genotypes that were identified from a shared cohort for any pair-wise comparison. The equation for this analysis is the following: % Common signature loci = ((x–z) / ((y_1_ + y_2_)–z)) *100; where *x* = signature loci shared in both cancers; *y_1_* = total signature loci in cancer1 (i.e. BC, GBM, LGG, or MEL); *y_2_* = total signature loci in cancer2 (i.e. GBM, LGG, MEL, or MB); *z* = CAML common to both cancers.

To further identify tissue specificity and disease-linked loci, we calculated the proportion of non-signature CAML genotypes shared between two cancers from the total shared loci (signature plus non-signature loci). Non-signature loci are those that pass statistical significance tests, but fail false discovery tests so are considered potentially informative, and may attain a higher level of significance when studies are verified with larger number of samples. Here, we hypothesized that the proportion of shared non-signature loci could demonstrate the degree of relatedness or non-relatedness between two tissues. Supporting this hypothesis, we discovered that from the total shared significant variant loci in BC and GBM, 47% were non-signature loci, described in Tables 1 and 3. Similarly, between BC and MEL, 42% of loci were non-signature. While, most of the loci shared between MEL and the brain cancers were above 79% signature CAML genotypes. Similarly, between the brain cancers (adult and childhood), the numbers of non-signature loci shared are relatively similar between any pair-wise comparisons (see Table 3).

**Table 3:** Non-Signature Variant Microsatellite Loci Shared by Cancers

Percentage of Shared Non-Signature Variant Microsatellite Loci					
	BC	GBM	LGG	MB	MEL
**BC**	-	47%	36%	20%	42%
**GBM**	-	-	41%	38%	13%
**LGG**	-	-	-	31%	21%
**MB**	-	-	-	-	7%
**MEL**	-	-	-	-	-

Described are the total microsatellite loci genotypes shared between any pair-wise comparisons of cancers and the proportion of shared loci that were not a part of disease signatures. The highest percentage of non-signature loci shared between two diseases was observed between BC and GBM; whereas, loci shared by MEL and MB were mostly CAML genotypes (93%). Equation: *% of Shared Non-Signature Variant Microsatellite Loci Shared = (x/y)* * *100*; *x* = non-signature loci shared by both cancers; *y* = total number of shared loci (non-signature plus signature loci) in a comparison.

To determine the biological significance of shared genotypes, we reviewed genes harboring loci and determined which implicated genes were shared by the described cancer populations. From IPA analysis, we discovered that polyamine regulation associated with colon cancer was the most common and significant (*p* < 0.01) pathway effected in any combinatorial analysis of the NTC/NCC lineage tumors. Additionally, the most common networks disrupted by genes associated with these MST loci were important to hereditary diseases and neurological disorders (described in Table 4). Comparisons with BC showed metabolic pathways as the most common networks disrupted and Ca^2+^ regulation as the most common pathway affected (see Table 5).

**Table 4:** Common Disease and Gene Functions for CAML genotypes shared between cancers

Common Cell Pathway Linked to Genes with CAML Genotypes Shared by Cancers from NTC/NCC Lineage		
Disease Comparisons	*p*-value	Canonical Cancer Pathway
GBM-LGG-MB	2.3 × 10^−3^	Polyamine Regulation
MEL-LGG-MB	2.3 × 10^−3^	
MEL-LGG-GBM	4.6 × 10^−3^	

Described is the *p*-value (*p* < 0.01 are significant) from comparisons between the cancers arising from tissue originating from NTC/NCC lineage. The most significant cell signaling pathway that was affected was colon cancer associated polyamine regulation. The gene commonly shared in these comparisons was *PSME*. Comparison of MEL-LGG highlighted polyamine regulation but was below the cut-off for significance.

**Table 5:** Shared Cell Pathways and Cellular Processes Linked to Genes with CAML

Common Cell Pathways Linked to Genes with CAML Genotypes Shared by BC & NTC/NCC Lineage Cancers			
Disease Comparisons	*p*-value	Canonical Pathway	Top Networks
BC-GBM	-	-	Carbohydrate Metabolism, Small Molecule Biochemistry, Cardiovascular Disease
BC-LGG	8.0 × 10^−3^	VDR/RXR Activation TR/RXR Activation	DNA Replication, Recombination, and Repair, Energy Production, Nucleic Acid Metabolism
BC-MB	3.2 × 10^−3^	Regulation by Calpain Protease	Lipid Metabolism, Small Molecule Biochemistry, Cellular Movement
BC-MEL	1.3 × 10^−2^	Regulation by Calpain Protease	DNA Replication, Recombination, and Repair, Energy Production, Nucleic Acid Metabolism
	1.6 × 10^−2^	VDR/RXR Activation TR/RXR Activation	

Genes with functions important to Ca^2+^ transport and regulation were identified with BC and LGG, MB, or MEL; no pathways were identified with GBM. The comparison between BC-MEL was not significant though similar to the other pair-wise comparisons described. Multiple networks were described with these associations with small molecule biochemistry, nucleic acid metabolism, and energy production being the most common. *P*-value describes significance with (*p* < 0.01).

## DISCUSSION

With the reoccurrence of most cancers diagnosed at advanced stages and following chemotherapeutic and radiological treatments; and with more than 50% of somatic mutations arising prior to tumor formations in several cancers, efforts to identify cancer cell(s) of origin that are tissue-specific are intensively studied [22–24]. Genomic variants in non-coding regions of genomic material, including those in microsatellites, are accelerating the identification of cancer-promoting elements which may be additive to the effects of mutations in the coding regions of genes. These data show CAML genotypes, specifically those identified in melanoma (an NCC lineage tissue) were pervasive in the brain cancers (NTC lineage tissue) (Tables 1 and 2). This suggests that the spectrum of CAML genotypes in MEL may be attributed to variants in embryonic ectodermal tissues which might contribute to tumors with NCC and/or NTC lineage. As example, *LAMP1* (a gene with an intronic CAML discovered in MEL, GBM, LGG, and MB) is identified with melanoma metastasis to lung tissue and notable *LAMP1* expression on the cell membrane of astrocytomas was recently discovered in immunohistochemistry analysis [25, 26]. Further supporting this tissue lineage association, advanced melanomas frequently metastasize to the brain and advanced neuroblastoma patients are commonly also susceptible to skin cancers [1].

We found relationships with GBM to be revealing, given that there were several CAML genotypes shared between GBM and all of the cancers (including BC). Identifying disease specific markers is challenging and especially with GBM, as also recently demonstrated by a single-cell sequencing analysis of GBM tumors which discovered high genomic variability between cells and RNAs [27]. Thus, finding comparable numbers of CAML between GBM vs BC and GBM vs LGG may further add to biological distinctions between GBM from other glioma types or grades.

Among genes shared with BC and NTC/NCC lineage cancers, in this study, *NCOR1* was an important discovery. *NCOR1*, *NCOR2* and *HDAC3* collectively form the N-CoR transcription co-repressor complex [28]. Thus, modifications to *NCOR1* could lead to changes in gene expression [28, 29]. Additionally, we discovered variants in *KIF1B* and *TLN2* in BC and shared with NTC/NCC lineage cancers: isoform-2 of *KIF1B* is important for neuronal apoptosis [30]; and *TLN2* has been monitored in cerebrospinal fluid of epileptic patients and is mostly known for its contribution during plaque formation in cytoskeletal interaction with integrins [31]. Therefore, we wonder if these microsatellite variants may be important towards identifying potential transcriptional variants of genes that are preserved in tissue-specific tumors but are otherwise additive to tumorigenesis across numerous tissue types. Separately, BC can metastasize to brain tissues thus these shared genotypes, notably, between GBM and BC allow us to speculate as to whether such loci potentiate or may be sensitive predictive indicators of BC metastasis to brain tissue; although GBM and cancer metastasis to brain tissue do show different disease pathology. Potentially supportive of this nascent hypothesis are recent data showing modified expression and metabolic activity by *GLUD1/2* in gliomas with *IDH1* mutation, we identified an intronic variant in *GLUD1* in our GBM CAML signature and this locus is shared with BC (although, in BC the locus is a non-signature variant) [32, 33]. Thus, CAML genotypes shared by breast cancer and the NTC/NCC lineage cancers may be generally important to cancers or indicate metastatic potential. Additionally, those genes associated with BC and the NTC/NCC lineage cancers show calcium regulatory pathways to be frequently shared (see Table 4). Calcium regulation is important during cell cycle and mobility and is observed to be a cell-division signal exploited by tumors [34, 35]. And, in neural tissues Ca^2+^ release provides signal transduction and promotes neural cell elongation, processes notably exploited in the tumor microenvironment [36].

Thus, variants identified through our study could introduce tissue and disease specificity (as demonstrated by the uniqueness of some CAML signatures to an explicit pathology versus those shared between diseases (i.e. MEL vs. LGG)) suggesting the potential for conserved biology that could extend our understanding of cancer etiology. As such, the biological associations to hereditary and neurological diseases and disorders among genes containing overlapping CAML genotypes in the NTC/NCC lineage cancers suggests that modification to non-coding, intronic regions are (1) sensitive to disease manifestation and, (2) differing combinations of tissue-specific CAML genotypes may contribute to diseases of NTC/NCC lineage, albeit different disease phenotypes, due to alternative splicing via non-coding variants [37]. Accordingly, a cancer-associated variant was identified in an intron of *PSME* in all of the cancers linked to NTC/NCC lineage (MEL, GBM, LGG, and MB); PSME is a proteasome activator that promotes *MDM2* dependent degradation of *p53*, preventing apoptosis after DNA damage. This conserved variant in all NTC/NCC originating cancers suggests the possibility to identify tumors with wild-type *p53* that are regulated differently due to CAML variants in *PSME* [38]. Furthermore, the identification of a variant locus in *FUBP3* in all the brain cancers (GBM, LGG, and MB) supports the potential for tissue-specific CAML and disease linkage. *FUBP3* has previously been shown to regulate the expression of *FGF9*, a gene important during embryogenesis and healthy neuronal cell differentiation and development [39–41]; FGF9 also contributes to gliomagenesis [42, 43]. Interestingly, FUBP3 binds to a microsatellite repeat region at the 3′-end of *FGF9* and regulates its expression [40]. Thus, we speculate whether *FGF9* regulation could be modified due to CAML genotypes in *FUBP3* and importantly in brain cancer pathogenesis. As previously identified, mutations in *FUBP1* and *IDH1* are closely associated with oligodendrogliomas, and are important prognostic and molecular markers for differentiating glioma phenotypes [24, 44, 45].

## MATERIALS AND METHODS

### Microsatellite genotyping

Exome sequencing data, from Illumina HiSeq sequencing machines were obtained from The Cancer Genome Atlas (TCGA) and the 1000 Genomes Project (1kGP). Sequences from ethnically matched cohorts were used in these analyses, which included Caucasian populations for both cancer and control groups. Sequences were aligned to a reference human genome (hg19) using BWA, and MST loci were identified with methods previously developed by our laboratory [10, 15, 16]. Loci with sequencing reads with a depth of coverage 15x or greater were used in these analyses for uniformity of sequenced data quality. A population of alleles from cancer genomes (TCGA data) and control (non-cancer samples; 1kGP) cohorts was created. An allele is defined by a genomic locus with a specific microsatellite repeat and nucleotide sequence length; in each sample a pair of alleles was identified and each pair was defined as a genotype. The most common genotype(s) for a locus was identified in control (1kGP) samples; this genotype was defined as the consensus or pre-dominant genotype (if more than a pair of alleles was identified for a locus then that locus in that sample was not used). Similar to the analysis of 1kGP samples, glioblastoma (GBM), lower-grade glioma (LGG), medulloblastoma (MB), and melanoma (MEL) samples were analyzed for genotypes, loci with significantly different variants (non-predominant) from the consensus (predominant genotype) in one population compared with the second population were identified as significant (*p*-value ≤ 0.01). The statistically significant genotypes were determined from data adjusted for false discovery rate (FDR), using a two-sided Fisher's exact- test and Benjamini-Hochberg correction and these genotypes were assembled into a signature cohort.

More specifically, an R script computed the adjusted *p*-value for each locus using the two-sided Fisher-test function. The Benjamini-Hochberg cut-off was selected as 0.01% (computed as the FDR < 1/X (where X is the total number of loci with *p*-value < 1 for the signature)) to reduce the identification of false positives. Those genotypes, that were individually significant and informative, were then assembled into the described ‘signature’ or a collection of cancer-associated microsatellite loci (CAML) which together increase the statistical significance across all samples; loci that were significant but did not pass FDR correction compose a non-signature set. Relative risk for each locus was computed as the percent of individuals with the non-predominant genotype from the cancer set divided by the percent of individuals with the non-predominant genotype in the control set. Sequences included 390 (*n* = 249 female; *n* = 141 male) control samples from the 1kGP, GBM germline (*n* = 252), LGG germline (*n* = 136), melanoma germline (MEL; *n* = 149), and breast cancer germline (BC; *n* = 656) through the Cancer Genomics Hub (CGHub) (dbGAP Study Accession: phs000178.v8.p7) [17], and medulloblastoma (MB; *n* = 90; dbGAP Study Accession: phs000504.v1.p1). These samples, like all others, were processed to remove any reads that did not meet the QC thresholds required in the 1kGP [18]. Next, we created a microsatellite target set: initially a population of over 1 million MST loci was identified in the human genome (NCBI36/hg18) using Tandem Repeats Finder (TRF) [19], using established methods [11, 14]. These data were filtered using a custom Perl script with SAMTOOLS [20] with specific parameters important to the flanking sequences used to identify loci, repeat region nucleotide length maxima and minima, SNP variations, and InDELs [11, 14]. Loci were further identified using RefSeq data from the UCSC Genome Table Browser [21]. As previously described, our methods resulted in 96.5% validation of identified MST genotypes from Mendelian inheritance of triads, Sanger sequencing data and HapMAP; these data are described in a publically available database (http://discovery.vbi.vt.edu/MicrosatDB/) [10].

### CAML overlap analysis between cancers

A custom R script was used to identify statistical differences (*p* < 0.05) between any three cancers. For this analysis, data were measured using a 3-set overlap comparison and organized according to the Venn diagram shown in Supplementary Figure S1 (Supplementary Figure S1). A one-sided Fisher's exact test was used to determine significance and demonstrate the extent of overlap; see Supplementary Figure S1.

### CAML gene function analysis

Using Ingenuity Pathway Analysis Systems^©^ (Qiagen, Inc), comparisons between genes with CAML genotypes shared among the five cancers were conducted to identify enriched gene functions, pathways, and diseases/disorders. Significant (*p* < 0.01) data are reported in Table 4.

## CONCLUSIONS

Variant cancer-associated microsatellite loci appear to demonstrate disease and tissue specificity [14]; identifying these variants from germline DNA highlights the potential for conserved cancer and tissue-specific mechanistic attributes and therapy targets in addition to understanding cancer origin. Locating CAML variants that are shared globally by tissues originating from ectodermal tissues (NTC and NCC: LGG, MB, GBM, and MEL) but also distinct to the central nervous system (GBM, LGG, and MB) and further unique from breast cancer (a non-nervous system tissue) strengthens the argument for cancer cells of origin being in-part intrinsic to the ecology of tumorigenesis, cancer, and the individual.

## SUPPLEMENTARY FIGURE AND TABLES




