# Supplementary material for: ‘Cut from the same cloth’: Shared microsatellite variants among cancers link to ectodermal tissues-neural tube and crest cells
Source: Oncotarget. 2015 Jun 17;6(26):22038–47. doi: 10.18632/oncotarget.4194 (PMC4673144; doi:10.18632/oncotarget.4194)
Supplement: Supplementary file 1 [file oncotarget-06-22038-s001.pdf]

SUPPLEMENTARY FIGURE AND TABLES

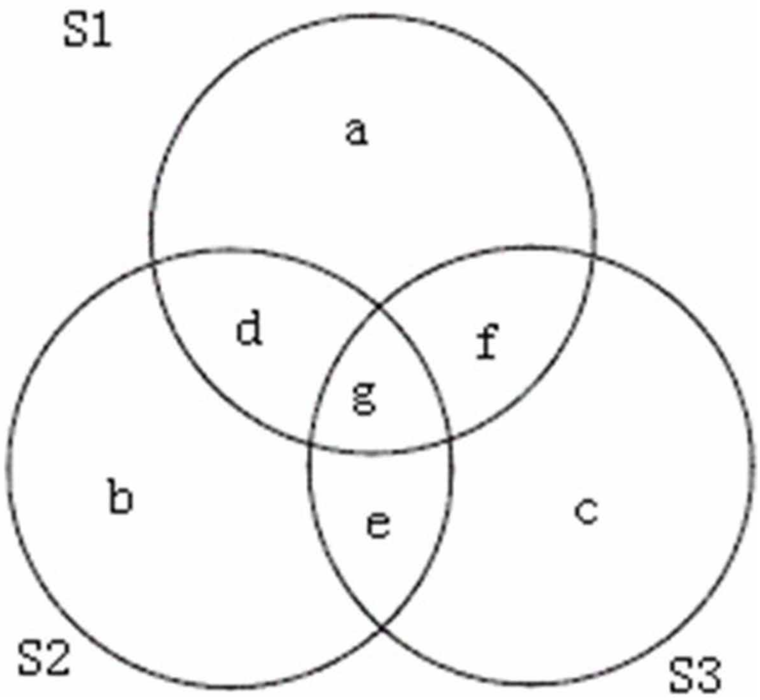

Supplementary Figure S1: CAML Overlap Test. Illustration of testing for the overlapping differences between S1 (i.e. Cancer 1) vs. S2 (i.e. Cancer 2) and S1 (i.e. Cancer 1) vs S3 (i.e. Cancer 3), using 7 counts from the partition of a 3-set Venn diagram; (as an example: S1 (LGG) vs S2 (GBM) and S1 (LGG) vs S3 (BC)). The resulting *p*-value is from a one-sided Fisher's test, indicating whether there is a significant difference between d + g (i.e. S1 vs S2) and f + g (i.e. S1 vs S3), given the following conditions (under the null hypothesis that no difference presents): (1) the overlapping count of d + g follows a hypergeometric distribution; (2) the overlapping count of f + g follows a hypergeometric distribution; (3) choosing S2 from (S1 union S2 union S3) and choosing S3 from (S1 union S2 union S3) are independent.

Supplementary Table S1: Shared Variant Microsatellite loci identified from between a pair-wise comparison of cancers. The first number describes the total loci shared and the second number identifies the total CAML genotypes shared by two cancers.

Total Number of Variant Microsatellite Loci / Total CAML Shared between Cancers

	BC	GBM	LGG	MB	MEL
BC	-	32/17	14/9	10/8	24/14
GBM	-	-	29/17	16/10	24/21
LGG	-	-	-	31/22	58/46
MB	-	-	-	-	30/28
MEL	-	-	-	-	-

Supplementary Table S2: Described are the total CAML genotypes shared between any pair-wise comparisons of cancers

Percentage of *Signature* Variant Microsatellite Loci from Total Shared

	BC	GBM	LGG	MB	MEL
BC	-	53%	64%	80%	58%
GBM	-	-	57%	63%	88%
LGG	-	-	-	71%	79%
MB	-	-	-	-	93%
MEL	-	-	-	-	-

Supplementary Table S3: Shared CAML Genotypes between Cancers from total shared loci (signature and non-signature). Described is the percentage of cancer-associated microsatellite loci identified in two cancers from a pair-wise comparison (Equation: *% shared CAML genotypes = ((x/y)*100))*; where x is the number of shared CAML by both cancers; y is total number of shared loci (signature and non-signature). The greatest population (38%) of shared CAML genotypes was identified in both LGG and MEL. The least number of CAML genotypes shared (7.0%) were between LGG and BC. A comparable percentage of CAML were consistently identifiable between all cancers and GBM. We also hypothesized that disease specificity may further filter CAML genotypes into disease specific cohorts, thus limiting the number of shared CAML genotypes. Our results indicate that most of the cancers share less than 20% of the same signature loci. However, GBM when compared with any of the cancers showed similar fractions of CAML genotypes in any comparison, including BC. LGG showed the greatest range when compared with other cancers 7% with BC and 38% with MEL.

Percentage of *Signature* Variant Microsatellite Loci Shared by Cancers

	BC	GBM	LGG	MB	MEL
BC	-	22%	7.0%	10%	13%
GBM	-	-	21%	25%	25%
LGG	-	-	-	13%	38%
MB	-	-	-	-	13%
MEL	-	-	-	-	-
